# Incidental Finding of Riedel’s Lobe of the Liver and Intrahepatic Cholangiocarcinoma

**DOI:** 10.7759/cureus.40683

**Published:** 2023-06-20

**Authors:** Rami Al-Handola, Justine Chinnappan, Mohammad Bakeer, Sarah Ayad

**Affiliations:** 1 Internal Medicine, Hurley Medical Center, Michigan State University College of Human Medicine, Flint, USA

**Keywords:** cholangiocarcinoma, hepatic tumors, liver morphology, accessory liver lobes, adenocarcinoma of the liver, riedel’s lobe

## Abstract

Riedel's lobe is a rare anatomical variant of the liver morphology with a downward tongue-like projection of the anterior edge of the right lobe. It is usually detected incidentally with abdominal imaging performed for other indications. We present a case where we found Riedel's lobe incidentally, with invasive adenocarcinoma arising from close proximity. A 64-year-old female came in with encephalopathy and was found to have a distended abdomen with tenderness. Imaging revealed a complex lobular mass in the right hepatic lobe with an elongated extension of the right lobe inferiorly consistent with Riedel's lobe. The differential considered was malignancy versus abscess. CT-guided biopsy revealed invasive adenocarcinoma consistent with intrahepatic cholangiocarcinoma, which was further supported by the presence of portocaval lymph nodes. Various studies have tried to establish an association between Riedel's lobe and the occurrence of cancer arising from the surrounding structures, primarily from liver or metastasis. This case report adds to the current literature when such an association is being studied.

## Introduction

Riedel’s liver lobe, the most well-known accessory lobe of the liver, is considered a rare anatomical variant of the liver morphology with a downward tongue-like projection of the anterior edge of the right lobe of the liver involving the segments V and VI [1,2]. It is usually detected incidentally with abdominal imaging performed for other indications and is asymptomatic. The clinical significance of this lobe lies in the fact that it can become symptomatic, causing abdominal discomfort, palpable right upper quadrant mass and rarely can present with serious complications such as torsion, especially when it is pedunculated. Other clinical significance includes its unclear association with cancer. These sites are thought to be considered foci for primary hepatocellular carcinoma or hidden metastasis. There is very little report about its association with intrahepatic cholangiocarcinoma. The knowledge of this variant is essential to not only prevent complications or diagnostic confusion, but the presence of a tumor in the vicinity of the lobe itself needs careful anatomical study when considering surgical resection of the tumor. The lobe can be visualized by imaging either with ultrasound, computed tomography (CT), or magnetic resonance imaging (MRI). We present a case of incidental finding of both Riedel’s lobe and invasive adenocarcinoma of the liver. This case was previously presented as an oral presentation at the 25th Annual Michigan State University (MSU) College of Human Medicine Community Research Forum, Flint, Michigan, on May 24, 2023.

## Case presentation

A 64-year-old female was brought to the emergency department due to altered mentation and fever. Her medical history includes asthma, hypertension, irritable bowel syndrome, sciatica, and arthritis. Her family history is remarkable for breast cancer in both her mother and sister. Her last breast and colon cancer screening with mammogram and colonoscopy were performed five years and four years ago, respectively, and were unremarkable. She does not have a personal history of cancer or liver conditions.

On presentation, she was hypertensive at 140/51, tachycardic at 105, tachypneic at 22, and febrile at 38.5. She was lethargic, oriented to self and location, and had a Glasgow Coma Scale (GCS) of 13. Examination revealed abdominal distension and tenderness in the right upper and lower quadrants without guarding or rigidity. Workup (Table 1) was significant for leukocytosis and positive COVID-19. Liver function tests and lipase were grossly unremarkable.

**Table 1:** Important laboratory tests obtained during the initial evaluation. WBCs: white blood cells; PCR: polymerase chain reaction; ALT: alanine aminotransferase; AST: aspartate aminotransferase.

Lab	Value	Reference Range
WBCs count	12.8 K/U	4.0-10.8 K/UL
COVID-19 PCR	Positive	Negative
Alkaline phosphatase	42 U/L	30-143 U/L
ALT	37 U/L	7-40 U/L
AST	42 U/L	0-40 U/L
Total bilirubin	1.2 mg/dL	0.3-1.2 mg/dL
Direct bilirubin	0.3 mg/dL	0.1-0.2 mg/dL
Lipase	61 U/L	0-58 U/L

**Figure 1:**
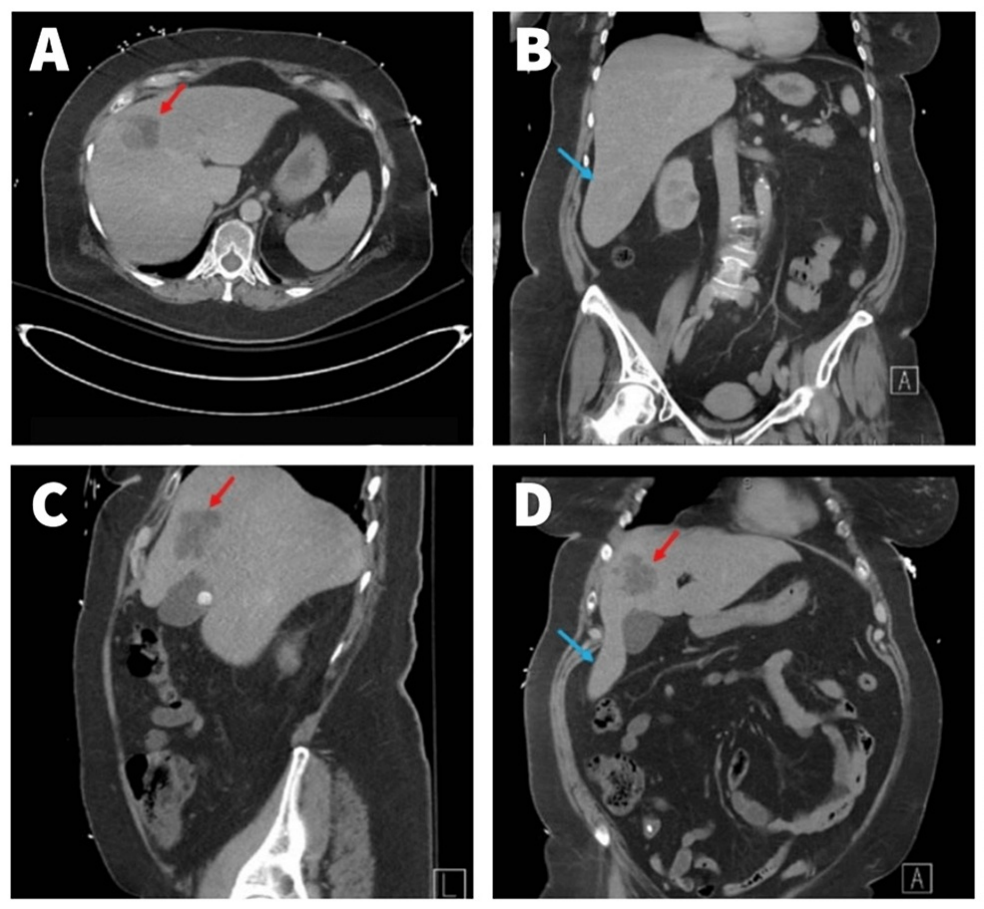
CT of the abdomen and pelvis. CT of the abdomen and pelvis showing lobulated mass within the left lobe of the liver (red arrows in A, C, and D) and tongue-like extension of the right lobe inferiorly (blue arrows in B and D). CT: computed tomography.

**Figure 2:**
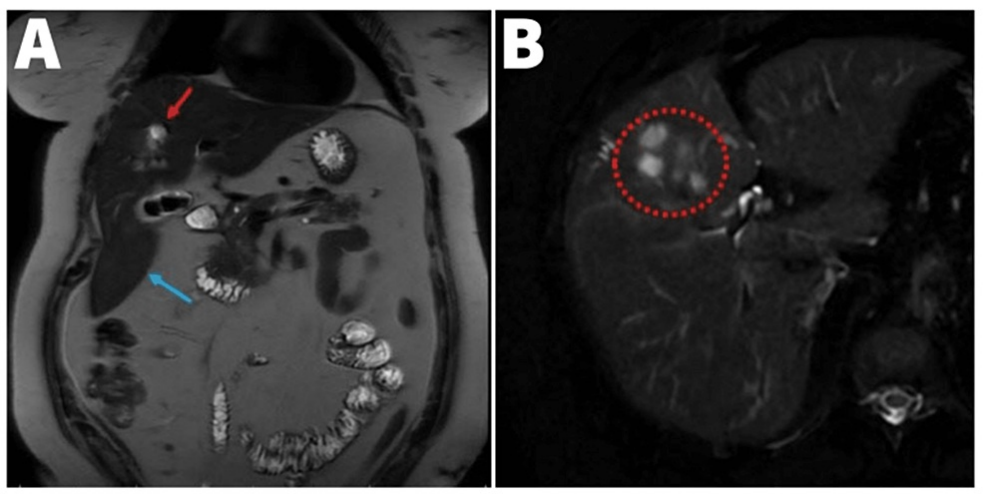
MRI of the abdomen and pelvis. MRI of the abdomen and pelvis (A) showing a complex appearing lobular mass (red arrow) measuring approximately 4.2 x 5.0 x 4.3 cm at the junction of the left and right lobe (segment IV to segment VIII) and elongated tongue-like extension of the right lobe of the liver inferiorly consistent with the Riedel's lobe (blue arrow) and (B) enlarged view of liver showing the complex appearing lobular mass (red dotted circle). MRI: magnetic resonance imaging.

Computed tomography (CT) without contrast of the head was normal. Given the abdominal tenderness, with fever and leukocytosis, she was further evaluated with CT of the abdomen and pelvis with contrast, revealing a lobulated mass within the right lobe of the liver measuring 3.9 cm (Figure 1) in addition to cholelithiasis. Subsequent magnetic resonance imaging (MRI) was obtained, which showed a complex appearing lobular mass measuring approximately 4.2 x 5.0 x 4.3 cm with a normal size of the liver in the superior border and an elongated extension of the right lobe of the liver inferiorly consistent with a Riedel's lobe (Figure 2) and mildly prominent portocaval region lymph node measuring 17 x 10 mm. Given the lobular mass on imaging along with her presentation, a differential diagnosis of malignant primary hepatic neoplasm or infectious etiology such as hepatic abscess was considered. A CT-guided biopsy and an aspirate of the surrounding fluid were obtained. Aerobic and anaerobic cultures of the aspirate did not yield growth. The histopathology revealed the liver tissue containing glandular neoplastic cells consistent with invasive adenocarcinoma. Immunohistochemical stains (Table 2) were positive for cytokeratin 7 and hepatocyte-specific antigen (HSA or HepPar-1), while negative for the other stains.

**Table 2:** Immunohistochemical stains results. HSA/HepPar-1: hepatocyte-specific antigen; CDX-2: caudal type homeobox 2; TTF-1: thyroid transcription factor; GATA3: GATA-Binding protein 3; PAX-8: paired-box gene 8.

Immunostains	Result
Cytokeratin 7	Positive
HSA (HepPar-1)	Positive
Cytokeratin 20	Negative
CDX-2	Negative
Napsin-A	Negative
TTF-1	Negative
GATA3	Negative
PAX-8	Negative
Arginase-1	Negative
Glypican 3	Negative

The primary site of origin of the adenocarcinoma is suspected to be from the pancreaticobiliary tree, including intrahepatic cholangiocarcinoma based on immunohistochemistry. Alpha-fetoprotein tumor marker was within the reference range with a value of 5.1 ng/mL (<9.0 ng/mL). The patient was treated with supplemental oxygen and dexamethasone initially for COVID-19 infection. Remdesivir was considered as well but deferred following the patient's refusal. She was also empirically treated with piperacillin/tazobactam for concerns of abdominal infection. The patient clinically improved and was weaned off oxygen. She was instructed to follow up with her primary care provider and oncologist for further evaluation and treatment of invasive adenocarcinoma of the liver likely arising from the pancreaticobiliary tree.

On follow-up two months later, she is asymptomatic and is following up with an oncologist. She underwent a positron emission tomography (PET) scan, which revealed 5.4 cm metabolically active in the right hepatic lobe mass. Her tumor markers, including carbohydrate antigen (CA 19-9) and carcinoembryonic antigen (CEA), were elevated at 11,876 and 14, respectively. The liver tumor board evaluated her, and given the multiloculated cystic lesion in the segment 4/8 with restricted diffusion or delayed enhancement, 1.3 cm portocaval lymph node, and histopathology finding, the tumor is consistent with intrahepatic cholangiocarcinoma. She is being considered for tumor resection in addition to chemotherapy with gemcitabine and cisplatin.

## Discussion

Riedel’s lobe of the liver, also known as the tongue lobe, is a rare anatomical variant described as an elongated extension of the right liver lobe that extends downward [1]. The incidence of Riedel’s lobe shows a significant variation, ranging from 3.3% to 31% [3]. This variability may be attributed to the lack of consistent criteria and the use of various diagnostics [3]. It is more commonly found in females than in males [2]. Knowledge about this variant is essential to prevent the development of complications and diagnostic confusion associated with this variation. The exact etiology of this anatomical variation remains unknown, and it is speculated to be either congenital or acquired in nature [1]. Intraperitoneal or intrapelvic inflammation is suspected to result in the acquired Riedel’s lobe in predisposed individuals [4]. Riedel himself thought the lobe was due to traction or gallbladder inflammation in prone individuals. Our patient was found to have cholelithiasis with no other source of inflammation, so the exact etiology of Riedel’s lobe in our patient is unknown.

In the majority of cases, Riedel’s lobe is asymptomatic and is often discovered during routine diagnostic and imaging studies. However, when symptoms do occur, patients may experience mild discomfort, abdominal tenderness, nausea, bloating, constipation, or distension, which can be attributed to the elongation of the lobe and the possible compression of nearby structures such as abdominal vasculature, stomach, and kidneys. These symptoms can occasionally mislead physicians to other diagnoses if imaging modalities are not obtained. One of the differential diagnoses of this anatomical variation is hepatomegaly; therefore, awareness of this variation is crucial to prevent the deceptive appearance of hepatomegaly [1]. Although our patient did not have a palpable abdominal mass, reports have noted palpable, enlarged livers that can be included within the differential of right-sided abdominal masses [1,3]. Various imaging modalities have been used to aid in the diagnosis, including ultrasound (US), CT, magnetic resonance imaging (MRI), and occasionally radionuclide imaging, as well as arteriographic examinations [1]. These investigations are essential to confirm the presence of this variation and possible complications and depict any other liver pathologies.

In general, asymptomatic and uncomplicated cases of Riedel’s lobe tend to have a favorable prognosis and do not require any specific management. However, mechanical complications have been reported with Riedel’s lobe, mostly related to the occurrence of torsion and extrinsic compression [2,3]. Thus, in symptomatic and complicated cases, management depends on the severity. Pain symptoms can be managed by analgesics, and surgical interventions are reserved for reasons such as chronic debilitating symptoms, life-threatening torsion, and additional pathologies within Riedel’s lobe, including but not limited to hydatid cysts and primary or metastatic cancers [5]. In our case, the patient had a follow-up with an oncologist, and the liver mass with histopathology of invasive adenocarcinoma was further identified to be intrahepatic cholangiocarcinoma and is planned for resection and chemotherapy.

While the association between Riedel’s lobe and hepatic tumors has not yet been clearly identified, the possibility of the chronic inflammation that is postulated to result in the Riedel lobe increasing the chance of cancer arising from the surrounding structure adds to the need for further studies. Our case adds to the limited available literature regarding Riedel’s lobe and concomitant invasive adenocarcinoma of the liver suspected to arise from the pancreaticobiliary tree.

## Conclusions

Riedel’s lobe is a rare anatomical variant of the liver with a downward projection of the anterior right lobe. It is usually discovered incidentally, has an asymptomatic presentation with a better prognosis than symptomatic patients, and requires no treatment. However, symptomatic and complicated cases will require further management, such as surgical resection of the lobe. The association between Riedel’s lobe and hepatic tumors has not yet been identified; the possibility of the chronic inflammation that is postulated to result in the Riedel’s lobe increasing the chance of cancer arising from the surrounding structure adds to the need for further studies.
